# Dentistry! What Is the Matter with You?

**Published:** 1921-04

**Authors:** Herbert G. Frankel

**Affiliations:** 12 W. 7th St., Cincinnati, O.


### Dentistry! What is the Matter with You?

By HERBERT G. FRANKEL, D.D.S.

We of the profession have often heard one of our brothers say, "I wish that I had never studied dentistry. The profession is all wrong and a man can not make the money in it that he can in a business or trade."

Again we have heard the wail, "The laity do not appreciate good dentistry and I am wasting my time and life."

Have you ever thought this and made the same remark? Is it the profession that should be blamed or the individual?

I believe that the individual is partly to blame and the colleges are to blame as well.

When a man enters the dental school, he is taught how to make plates, bridges, crowns, inlays, etc., but he is left entirely upon his own resources as to the salesmanship he should use in disposing of his art. In other words, he is taught workmanship, but not values.

How many are there of us who do wonderful, skillful work and yet get mediocre prices for the same?

Then, how many of the colleges teach their students to sell health rather than mechanics? Is it better to put a beautiful bridge in the mouth with devital teeth as abutments or to teach oral hygiene with removable or sanitary appliances?

Salesmanship in dentistry is as important as prosthesis, crown and bridge or any branch of the profession. Without it one can not expect to get the real worth for his labor. All big corporations have realized this and have adopted the plan of calling their salesmen to conferences, where classes in salesmanship are given.

I believe that a course in salesmanship is necessary in dentistry and should be a part of the curriculum of every first-class dental school.

Secondly, dentistry is a business as well as a profession and the dentist should have a business knowledge along with his professional knowledge.

How many dentists are losing a great deal of money, due to the fact that this side of their profession has been neglected? Mr. Bosworth is absolutely right in his convictions of business in dentistry, and it behooves us to try to correct the profession and the young men coming into the profession and set them right, so that they will not make the same mistakes and learn through bitter experience and regret the things that we have suffered.

I believe that a course in dental bookkeeping and accounting should be installed in our colleges and the young men taught the right way to run their businesses.

The colleges picture the wonderful opportunities and the pleasantries of the profession, but they fail to tell that "the first hundred years are the hardest" and that sometimes you will wear out several suits of clothes before you really get started.

Now as to the individual.

I could start off telling you as the Calamity Howlers would, that the advertiser was killing prices and that they could not compete against three-dollar crowns and ten-dollar plates, but that is not where the fault lies. We can not class the advertiser as a competitor—if we do, we have cheapened our value and it is time to quit.

LOOK OVER YOURSELF!

What kind of an office have you? What kind of an appearance do you make? What is your ability as a salesman? What kind of work do you do? Do you put the value of health before the value of the almighty dollar? Do you really and truly live up to the motto of "Do unto others as you would have them do unto you" in your relations with your patients?

Taking up the first question of the office. Is your office clean, bright and inviting? Is it a place where sanitary operations can be performed, or is it a dirty, dark, gloomy place, with no attempts at sanitation? Are you using modern equipment and appliances?

What about your personal appearance? Is it inviting or would it repel a patient? Are you thoughtful, courteous, kind, with a great deal of patience, or do you appear sloven, curt, cruel, impatient and lose your temper easily?

Can you convince your patients by salesmanship what is best for them and by skillful demonstrations show why your value is worth what you ask? Do you ask your patients what they would like to have or do you prescribe for them as a real professional man should?

Do you read dental journals? Do you follow the teachers of the profession? Are you interested in working out problems that confront the profession? Is your work the best that you can give, or is it hurry up and thrown together work? Are you careful and painstaking in your work, or do you hurry and slap it in? WOULD YOU BE WILLING TO HAVE IT PLACED IN YOUR OWN MOUTH?

Are you putting health before money in so far as to advise a patient not to have a bridge or appliance in the mouth if you really believed that it would not hold?

Do you explain the danger of infection from blind apical abscesses and recommend the radiograph? Do you instruct your patients how to brush their teeth properly?

Do you run a brushwheel over the teeth and call it a prophylaxis?

Are you treating your patients with the same regard that you would like to be treated?

If you are not doing these things you can not say that dentistry is to blame, IT'S YOU.

Dentistry, like any other profession, has undergone great changes. The things that were considered good form twenty-five years ago are passê today, and the same will be true in the next twenty-five years.

We must advance, and to advance we must watch ourselves.

Walt Mason says, "To be an optimist one must be a pessimist, because all deep thinkers are pessimists." If we are pessimists we will think about our faults and correct them, and we all have them, even you and I.

12 W. 7th St., Cincinnati, O.

